# Supplementary figures and images for: The non-template functions of helper virus RNAs create optimal replication conditions to enhance the proliferation of satellite RNAs
Source: PLoS Pathog. 2024 Apr 17;20(4):e1012174. doi: 10.1371/journal.ppat.1012174 (PMC11057728; doi:10.1371/journal.ppat.1012174)

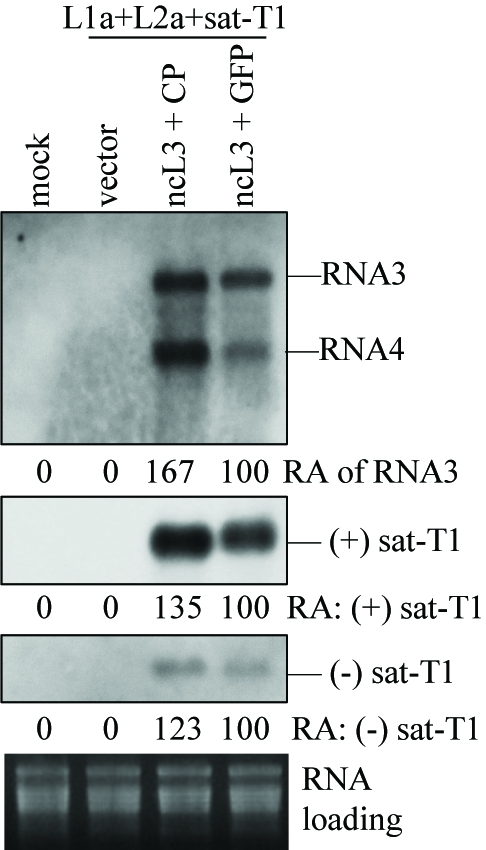

Supplement: S1 Fig — The 5th true leaves of N. benthamiana plants were infiltrated with Agrobacterium cells to express the replicase (L1a + L2a) and its noncoding RNA3 (ncL3) of LS-CMV, sat-T1, and either GFP or the CP protein of LS-CMV. In parallel, co-expression of the viral replicase and sat-T1 was used as the negative control. At 3 days post-agroinfiltration, total RNAs were extracted from the infiltrated leaves and subjected for RNA gel blotting analyses of ncRNA3, RNA4, and both polarities of sat-T1. The relative accumulation levels of these RNAs are shown below. Ethidium bromide-stained ribosomal RNAs were used for assessing the loading amounts of the RNA samples. (TIF) [file ppat.1012174.s001.tif]

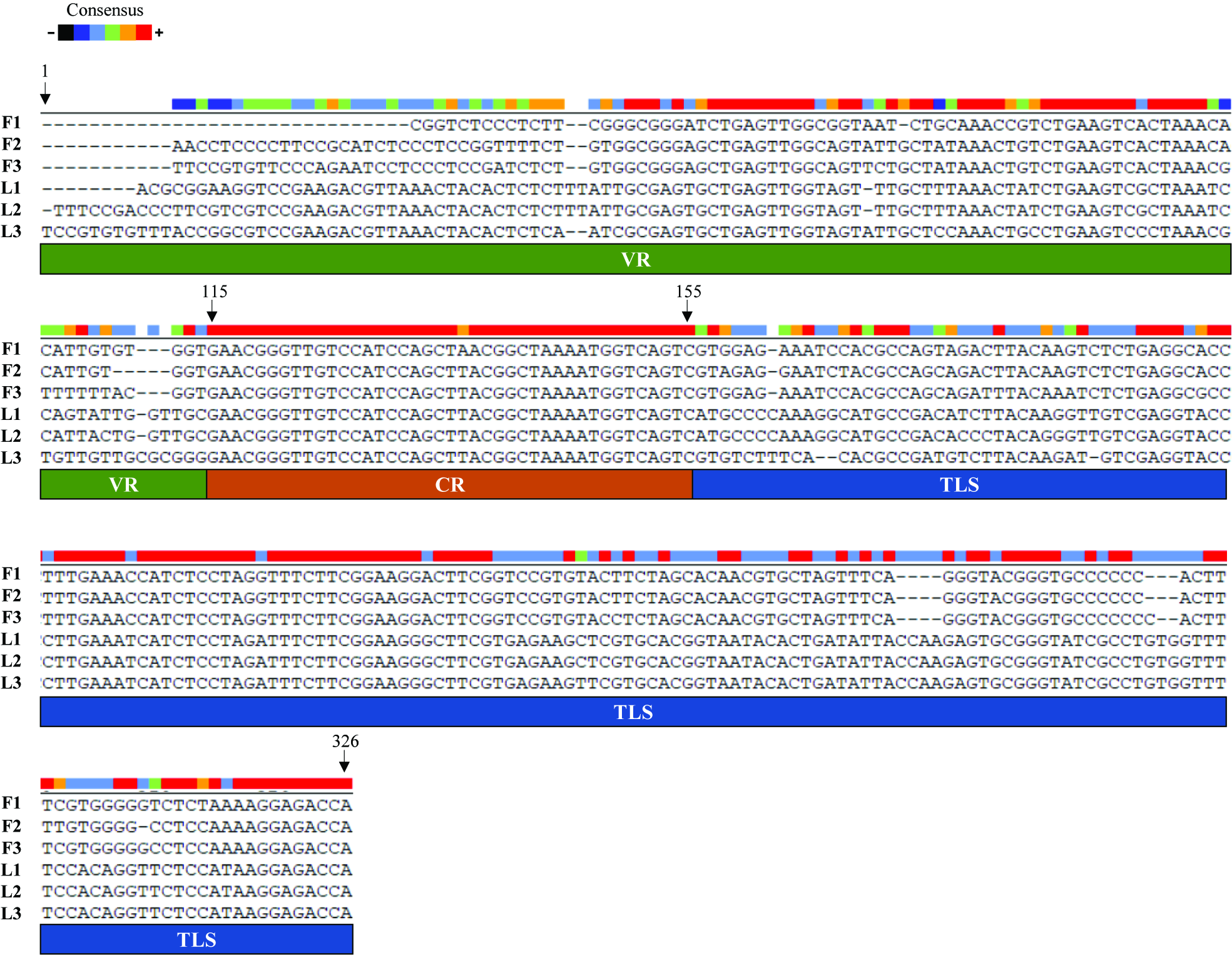

Supplement: S2 Fig — The sequence alignment was carried out using the MegAlign program in DNAstar. The 3′ UTR sequence is divided into three regions: a variable region (VR) at the 5′ end, a conserved tRNA-like structure (TLS) at the 3′ end, and a highly conserved stretch region (CR) separating them. (TIF) [file ppat.1012174.s002.tif]

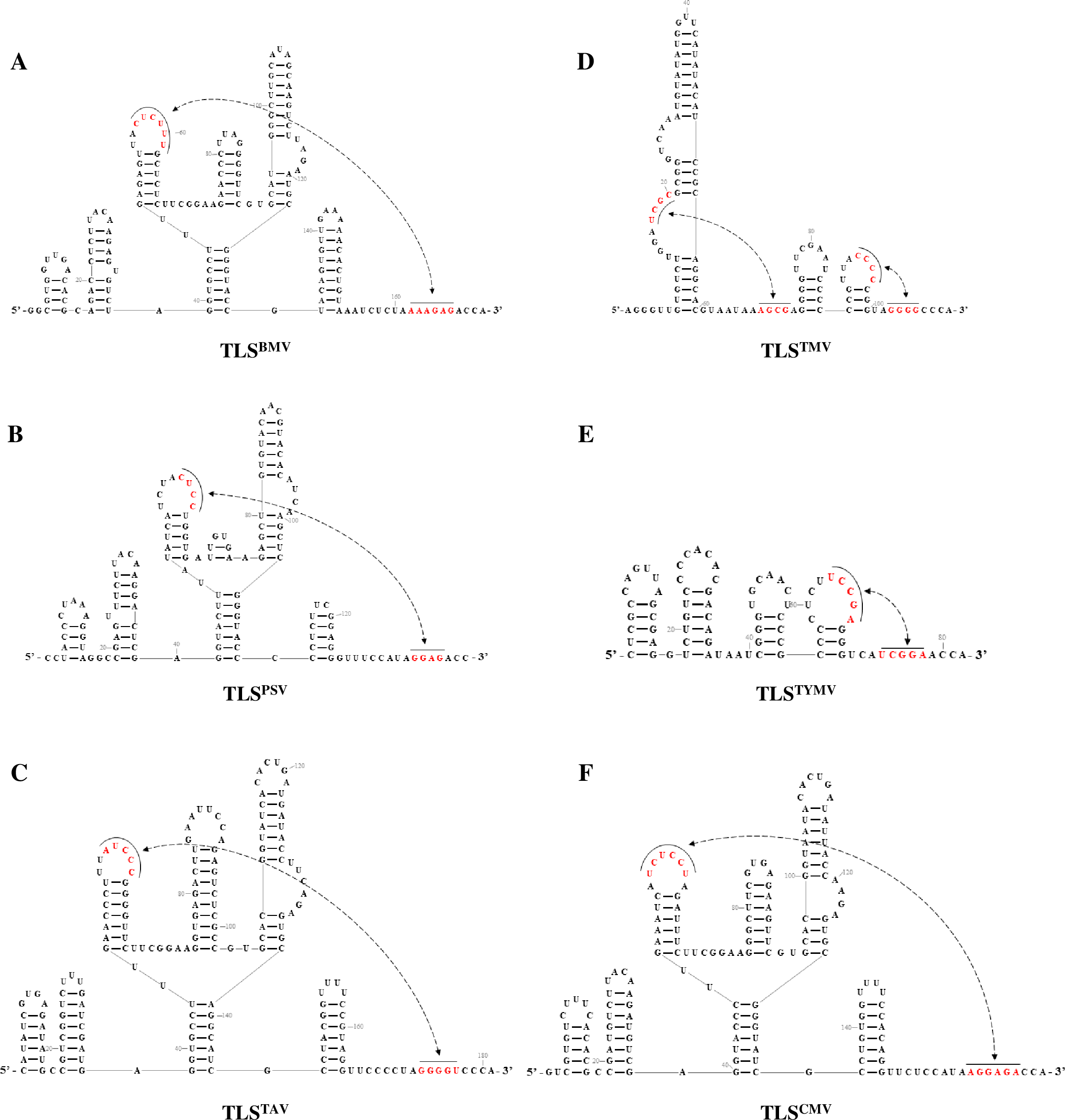

Supplement: S3 Fig — (A) TLSBMV: the TLS from brome mosaic virus (BMV) RNA3, (B) TLSPSV: the TLS from peanut stunt virus (PSV) RNA3. (C) TLSTAV: the TLS from tomato aspermy virus (TAV) RNA3. (D) TLSTMV: the TLS from tobacco mosaic virus (TMV). (E) TLSTYMV: the TLS from turnip yellow mosaic virus (TYMV). (F) TLSCMV: the TLS from RNA3 of cucumber mosaic virus LS strain. The RNA structures of TLSBMV, TLSTYMV and TLSTMV were reported previously [63–64]. The RNA structures of TLSPSV, TLSTAV and TLSCMV were predicted based on that of TLSBMV. All these structures were redrawn using RNA2Drawer (available at https://rna2drawer.app/). The dash lines with arrows indicate pseudoknots formed by the base-paring of the nucleotide sequences colored red. (TIF) [file ppat.1012174.s003.tif]

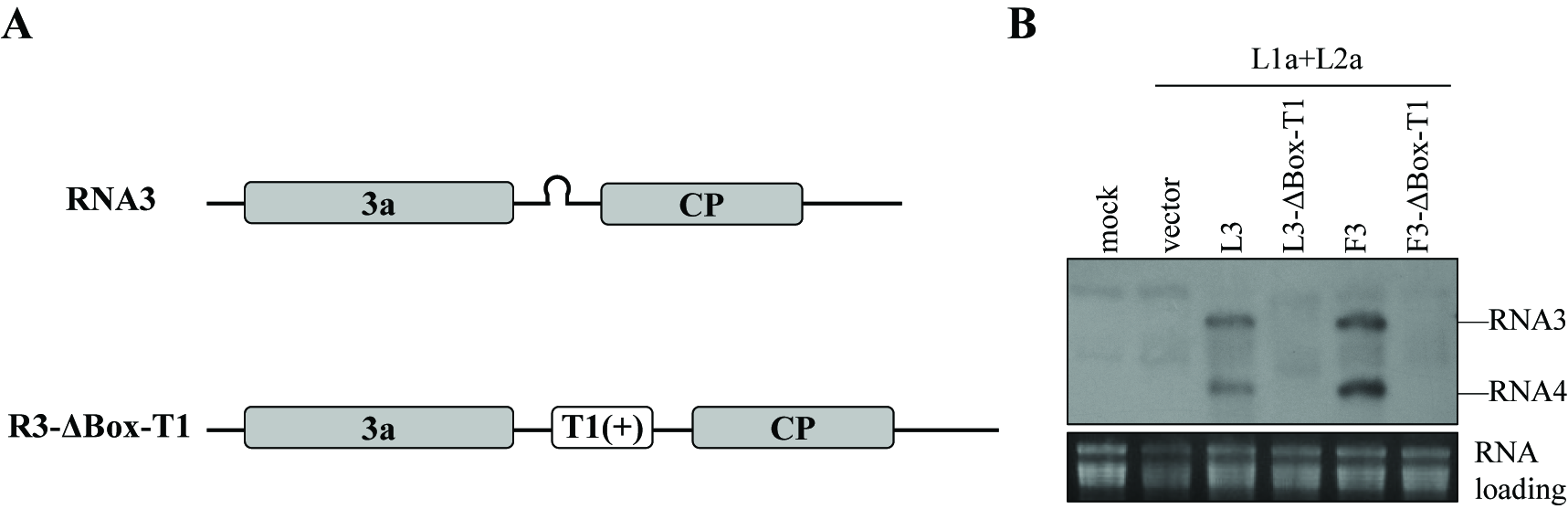

Supplement: S4 Fig — (A) Schematic diagrams of RNA3 and its derivative (R3-ΔBox-T1), in which the Box-B sequence was substituted with the (+) strand of sat-T1. (B) Northern blotting analyses of the accumulation of RNA3 and its variants. RNA3 from Fny-CMV (F3) or LS-CMV (L3), as well as their mutants or vector pCB301 was separately co-expressed with the replicase of LS-CMV, together with the RNA silencing suppressor P19. At 3 days post-agroinfiltration, total RNAs were extracted from the infiltrated leaves and subjected to northern blot hybridization. Mock plants were treated by infiltration solution alone. Ethidium bromide-stained ribosomal RNAs were used to assess the loading amounts of all RNA samples. (TIF) [file ppat.1012174.s004.tif]

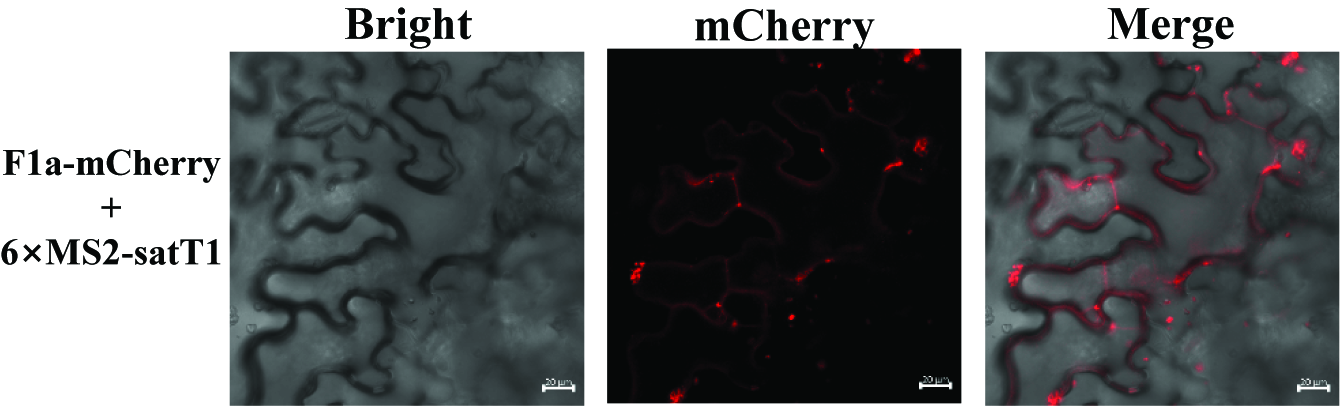

Supplement: S5 Fig — The lower epidermis of the leaves was infiltrated with Agrobacterium cells harboring the binary plasmids to express F1a-mCherry, 6×MS2-satT1, and p19. At 2 days post-agroinfiltration, the infiltrated leaves were subjected to Laser confocal microscopy for visualizing red fluorescence omitted from F1a-mCherry. (TIF) [file ppat.1012174.s005.tif]
