# Supplementary material for: The non-template functions of helper virus RNAs create optimal replication conditions to enhance the proliferation of satellite RNAs
Source: PLoS Pathog. 2024 Apr 17;20(4):e1012174. doi: 10.1371/journal.ppat.1012174 (PMC11057728; doi:10.1371/journal.ppat.1012174)
Supplement: S1 Table — (PDF) [file ppat.1012174.s006.pdf]

S1 Table. Primers used for constructing plasmids and strand-specific reverse transcription-polymerase chain reactions.

Names	Sequences	Purposes
ncF1a-F	CTGACGCAAGATCATCTTGAACGATCCACAACAGTTCG	To generate the plasmid pCB301-ncF1.
ncF1a-R	ATGATCTTGCGTCAGTTCATTAAGCGTTCCGCA	
ncF2a-1-F	TTTTCTACGGCTTTCCCTGCCCCCGCA	To generate the plasmid pCB301-ncF2. The pair of primers ncL2b-F/R are individually used to generate the plasmid pCB301-F2Δ2b.
ncF2a-1-R	GGAAAGCCGTAGAAAAGATACTAGAAAGAAAAGATTTAAGTCTAGG	
ncF2a-2-F	CCTGAAAGTGTTTGAAAGACCTGGTCGCGG	
ncF2a-2-R	CTTTCAAACACTTTCAGGTTCGGTGGGATGTTCTAG	
ncF2b-F1	TGTGATTGAACGTAGGTGCAACGACAAACGTCGAACTCC	
ncF2b-R1	CCTACGTTCAATCACATAATTCTTTCGCTGTTTGTTGG	
ncF2b-F2	CGTACGGTGGAGGCGAAGAAGCAGAGAC	
ncF2b-R2	CTCCACCGTACGAGCCAGTTGGAGTTCG	
ncF3a-F	CATGTAGTAGCTTTCCAAGGTACCAGTAGGACTT	To generate the plasmid pCB301-ncF3.
ncF3a-R	GGAAAGCTACTACATGCCTCGGGAAATCTAACAC	
ncF3b-F	TCATGTGATGACAAATCTGAATCAACCAGTGCTG	
ncF3b-R	GATTTGTCATCACATGACTCGACTCAATTCTACGAC	
ncL1a-F	ACGCTGAAAGATTGATGAGCTGACGCAAGGTCATTTTAAACGA	To generate the plasmid pCB301-ncL1.
ncL1a-R	ATCAATCTTTCAGCGTGACGTCACTTGTCACGAATACCCAAAC	
ncL2a-1-F	ACTATGACAAGTCCTCCACCCACTTTCTC	To generate the plasmid pCB301-ncL2. The pair of primers ncL2b-F/R are individually used to generate the plasmid pCB301-L2Δ2b.
ncL2a-1-R	GGACTTGTCATAGTAACAGAAAAGAGACTAGAAGT	
ncL2a-2-F	TGAGCTAGGACGTAGTTGTTGGCACCACT	
ncL2a-2-R	TACGTCCTAGCTCAAGCGACGCTGGA	
ncL2b-F	AGACAGTAGTGGTGTAGACCGCCGACCT	
ncL2b-R	TACACCACTACTGTCTACACATCCATTCTTCTTCTTCT	
ncL3a-F	TAGTAGCTTTCCAAGGTCCCAGTAGGACGTTAAC	To generate the plasmid pCB301-ncL3. The pair of primers ncL3b-F/R are also used in sgRNA4 to generate the plasmid pCB301-ncL4.
ncL3a-R	CCTTGGAAAGCTACTACATAACCTTCGTAATCTAGACAC	
ncL3b-F	TGATGACAAATCTGGATCTCCCAATGCTAGTAGAAC	
ncL3b-R	TCCAGATTTGTCATCACATAGGCACACTGAGAC	
L4-F	TTTGGAGAGGGTTTAGTTGTTCACCTGAGTCGTG	To generate the plasmid pCB301-L4
L4-R	CTAAACCCTCTCCAAATGAAATGAACTTCCTTATATAGAGGA	
L3ΔMP-F	ACGAAGGTTTGTTTTGTTACGTTGTACCTATGTATATATATACTACG	To generate the plasmid pCB301-L3-ΔMP and pCB301-L3-ΔCP, respectively. Both of two pairs of primers are used to generate the plasmid pCB301-L3-ΔMPΔCP.
L3ΔMP-R	CAAAACAAACCTTCGTAATCTAGACACGACACACTAA	
L3ΔCP-F	TGCCTTCCGTGTGTTTACCGGCGTC	
L3ΔCP-R	CACGGAAGGCACACTAAGACGCGAAAATAAAAA	
ncL3ΔVR-F	CGACTTAGGAACGGGTTGTCCATCCAGC	To generate the plasmid pCB301-ncL3-ΔVR.
ncL3ΔVR-R	CGTTCCTAAGTCGGGAGCATCCGTGAGATA	
ncL3ΔCR-F	CGCGGGGTCGTGTCTTTCACACGCCG	To generate the plasmid pCB301-ncL3-ΔCR.
ncL3ΔCR-R	GACCCCGCGCAACAACACGTTTAGGGACTTC	
ncL3ΔTLS-F	TGGTCAAAAAAAAAAAAAAAAAAAAAAAAAAAGGGTCGGCATGGC	To generate the plasmid pCB301-ncL3-ΔTLS.
ncL3ΔTLS-F	TTTTTTTTTTTTTTGACCATTTTAGCCGTAAGCTGGATGG	
ncL3ΔVRΔCR-F	ACTTAGGTCGTGTCTTTCACACGCCGATGTC	To generate the plasmid pCB301-ncL3-ΔVRΔCR.
ncL3ΔVRΔCR-R	ACACGACCTAAGTCGGGAGCATCCGTG	
TLS-F	GAGAGGGTCGTGTCTTTCACACGCCGATGT	To generate the plasmid pCB301-TLS
TLS-R	ACACGACCCTCTCCAAATGAAATGAACTTCCTTATATAGAG	
ncL3-3U-F	GGGTCGGCATGGCAT	To generate the vector without TLS^{CMV} for further LIC cloning.
ncL3-3U-R	ACTGACCATTTTAGCCGTAAGCT	
TAVTLS-F	GCTAAAATGGTCAGTCATATCGTGAGATATGCCG	To generate the plasmid pCB301-ncL3-TLS^{TAV}.
TAVTLS-R	ATGCCATGCCGACCCTGGGACCCCTAGGG	

TMVTLS-F	GCTAAAATGGTCAGTAGGGTTGTGTCTTGGA	To generate the plasmid pCB301-ncL3-TLS^{TMV}.
TMVTLS-R	ATGCCATGCCGACCCTGGGCCCCTACC	
BMVTLS-F	GCTAAAATGGTCAGTGGCGTGGTTGACAC	To generate the plasmid pCB301-ncL3-TLS^{BMV}.
BMVTLS-R	ATGCCATGCCGACCCTGGTCTCTTTTAGAGATTTACAGT	
PSVTLS-F	GCTAAAATGGTCAGTCCTACCCTAAAGGTAGGC	To generate the plasmid pCB301-ncL3-TLS^{PSV}.
PSVTLS-R	ATGCCATGCCGACCCTGGTCTCCTATGGAAACC	
TYMVTLS-F	GCTAAAATGGTCAGTCTCGCCAGTTAGCGA	To generate the plasmid pCB301-ncL3-TLS^{TYMV}.
TYMVTLS-R	ATGCCATGCCGACCCTGGTTCCGATGACCC	
ncL3Δ5UM13R-F	GGATAACAATTTCACACAGGAATGTAGTAGCTTTCCAAGGTC	To generate the plasmid pCB301-ncL3-Δ5UΔVRΔCR.
ncL3Δ5UM13R-R	GTGTGAAATTGTTATCCGCTCCCTCTCCAAATGAAATGAACTTC	
L3Δ5U II-F	CACGCATGGCTTTCCAAGGTACCAGTAGG	To generate the plasmid pCB301-L3-Δ5U II.
L3Δ5U II-R	AGCCATGCGTGGACTGACGCGACAC	
L3ΔBoxB-F	TTAATTTTGCTCCCTGTTGGGCCCCTTACT	To generate the plasmid pCB301-L3-ΔBoxB.
L3ΔBoxB-R	CAGGGAGCAAAATTAAAACTGTGATGTGGACTTAC	
L3mSLC-F	TAAGTCACTATTACCAAGAGTGCGGGTATCGC	To generate the plasmid pCB301-L3-mSLC.
L3mSLC-R	TCTTGGTAATAGTGACTTATTACCGTGCACGAAC	
F1a-*BamH*I-F	AATggatccATGGCGACGTCCTCG	To generate the plasmid pBI121-F1a-mCherry and pBI121-L1a-mCherry.
F1a-*Sac*I*Kpn*I-R	AATgagctcTTggtaccAGCACGAGCAACACATTC	
L1a-*BamH*I-F	AATggatccATGGCAACGTCCTCATTC	
L1a-*Kpn*I-R	AATggtaccGACTAACGGAATACAAGTAG	
mCherry-*Kpn*I-F	AATggtaccATGGTGAGCAAGGGC	
mCherry-*Sac*I-R	AATgagctcCTACTTGTACAGCTCGTCC	
T1-*BamH*I-F	AATggatccGTTTTGTTTGATGGAGAATTGC	To amplify the sequence of (+)-sense RNA of sat-T1 and a 337-nt DNA fragment of *GUS* gene for generating the plasmid pBI121-6×MS2-T1 and pBI121-6×MS2-gus.
T1-*Sac*I-R	AATgagctcGGGTCCTGTAGAGGAATG	
gus-*BamH*I-F	AATggatccGTTTTGTTTGATGGAGAATTGC	
gus-*Sac*I-R	AATgagctcTTCAAATGGCGTATAGCCG	
YFP-*Nco*I-F	AATccatggTGAGCAAGGG	To amplify the coding sequence of *yfp* gene for generating the plasmid pCAMBIA-MS2CP-YFP-NLS.
YFP-*Sac*I-R	AATgagctcTACTTCGTCTTCGACGT	
mF3-F	TGATTCCATTCCAATCTTACTCCCTGTTGAGCC	To generate the plasmid mF3 with the mutated Box-B motif.
mF3-R	GATTGGAATGGAATCAAAACTATGATGTAGGCTTACTAAAAC	
F3-LIC-F	TTCCGTGTTCCCAGAATCC	To create a gap between CP ORF and 3' UTR in the plasmid of both F3 and mF3, and generate the vector for further LIC cloning.
F3-LIC-R	TCAGACTGGGAGCACTC	
F3-gus-LIC-F	GTGCTCCCAGTCTGATTACGTCCTGTAGAAACCC	To amplify a part of sequence in *GUS* gene with the connector sequences for generating the plasmid of both pCB301-F3-gus and pCB301-mF3-gus.
F3-gus-LIC-R	TCTGGGAACACGGAAATGGCGTATAGCCGC	
mF3-T1(+)-LIC-F	GTGCTCCCAGTCTGAGTTTTGTTTGATGGAGAATTGC	To amplify the total sequence of (+)-sense RNA of sat-T1 with the connector sequences for generating the plasmid pCB301-mF3-T1(+).
mF3-T1(+)-LIC-R	TCTGGGAACACGGAAGGGTCCTGTAGAGGAAT	
mF3-T1(-)-LIC-F	GTGCTCCCAGTCTGAGGGTCCTGTAGAGGAAT	To amplify the total sequence of (-)-sense RNA of sat-T1 with the connector sequences for generating the plasmid pCB301-mF3-T1(-).
mF3-T1(-)-LIC-R	TCTGGGAACACGGAAGTTTTGTTTGATGGAGAATTGC	
F3ΔBox-LIC-F	TCTTACTCCCTGTTGAGC	To amplify the total sequence of Fny-RNA3 with the deletion of Box-B motif.
F3ΔBox-LIC-R	TCAAAACTATGATGTAGGCTTACT	
F3ΔBox-T1(+)-LIC-F	ACATCATAGTTTTGAGTTTTGTTTGATGGAGAATTGC	To amplify the total sequence of (+)-sense RNA of sat-T1 with the connector sequences for generating the pCB301-F3ΔBox-T1.
F3ΔBox-T1(+)-LIC-R	CAACAGGGAGTAAGAGGGTCCTGTAGAGGAAT	
L3ΔBox-LIC-F	TTTTGCTCCCTGTTGG	To amplify the total sequence of LS-RNA3 with the deletion of Box-B motif.
L3ΔBox-LIC-R	TTAAAACTGTGATGTGGACTTAC	
L3ΔBox-T1(+)-LIC-F	ACATCACAGTTTTAAGTTTTGTTTGATGGAGAATTGC	To amplify the total sequence of (+)-sense RNA of sat-T1 with the connector sequences for generating the pCB301-L3ΔBox-T1.
L3ΔBox-T1(+)-LIC-R	CAACAGGGAGCAAAAGGGTCCTGTAGAGGAATG	
sat-P2C-F	GCGCCCATGGTTTGCGCTTACCGTGGAAATTTCGAAAGAAAC	To generate the plasmid of the satellite mutant pCB301-P-2C.
sat-P2C-R	CCATGGGCGCGGTTCTGCTAGCAAACTGAG	

ncL3a-F	TAGTAGCTTTCCAAGGTCCCAGTAGGACGTTAAC	Using in strand-specific RT-PCR for detecting the synthesis of negative-strands.
ncL3b-R	TCCAGATTTGTCATCACATAGGCACACTGAGAC	

*The restriction enzyme sites are underlined.
