# Supplementary material for: The non-template functions of helper virus RNAs create optimal replication conditions to enhance the proliferation of satellite RNAs
Source: PLoS Pathog. 2024 Apr 17;20(4):e1012174. doi: 10.1371/journal.ppat.1012174 (PMC11057728; doi:10.1371/journal.ppat.1012174)
Supplement: Attachment — Submitted filename: R2-Responses to comments.docx [file ppat.1012174.s010.docx]

RESPONSES TO THE REVIEWER’S COMMENTS

Reviewer #2: I will encourage the authors to polish their english. Some sentences such as line 290-291 are unclear.

RE: Thank you. The whole manuscript has been polished by a native English speaker, and the changes are tracked in the revised version. The sentence mention here is corrected to “The difference in the accumulation levels of these viral RNAs produced by both viral replicases would be due to the distinct replication efficiency, as the RNA silencing suppressor p19 was included to suppress antiviral RNA silencing in this assay.” (Line 287-290).

The conclusion is line 384 needs to be edited. Isn’t the accumulation of negative strand stat 1 (-) RNA the evidence for sat-1 replication? If so, the conclusion seems incorrect.

RE: Thanks for pointing out the issue. The conclusion has been corrected to “These results suggest that while the coding sequences of both MP and CP are important for the synthesis of (-) sat-T1, the presence of either one is sufficient for L3 to facilitate sat-T1 replication. ”.

Could the accumulation of sat1(+) could be increase of stability of the RNA.

RE: It could be, and I have kept it in my mind. If possible, we will have a test in our future work.
