# Supplementary material for: The non-template functions of helper virus RNAs create optimal replication conditions to enhance the proliferation of satellite RNAs
Source: PLoS Pathog. 2024 Apr 17;20(4):e1012174. doi: 10.1371/journal.ppat.1012174 (PMC11057728; doi:10.1371/journal.ppat.1012174)
Supplement: Attachment — Submitted filename: Response to Reviewer comments.docx [file ppat.1012174.s009.docx]

**RESPONSES TO REVIEWER’S COMMENTS POINT-BY POINT BELOW**

Reviewer's Responses to Questions

**Part I - Summary**
Please use this section to discuss strengths/weaknesses of study, novelty/significance, general execution and scholarship.

Reviewer #1: This manuscript (no. D-23-01799) is quite interesting and novel in the way the system was examined. However, there are a number of corrections that need to be made, including some incorrect statements. These are listed further below with numerals. In addition, I think the authors need to consider more of the vast literature on this issue. Several points to consider are as follows:

**RE:** Thank you for the positive feedback on this work. We have throughout considered more literatures and made corrections and changes according to your comments as below.

A. The model look very much like the BMV replication model, where the 1a protein of BMV is produced in larger amounts than the 2a protein. That is not the case with Fny-CMV. There is always more 2a than 1a at steady-state level. In fact, in protoplast infected by Fny-CMV, the 1a protein is not even detectable by immune-blotting, although it can be detected in plant extracts. [See Virology (1995) 208: 58-66.] Thus, it is unlikely that there is excess 1a protein around to line the invaginated tonoplast membrane pocket depicted in Fig. 9. To this point, see also Discussion in Virology (2009) 383: 248-260. [Also, BMV replicates on the ER, a different membrane from CMV.]

**RE:** Thank you very much for bringing this error to our attention. It was indeed due to our ignorance. In the course of our experiments, we also noted that in plant tissues infected with CMV, RNA2 consistently accumulates more than RNA1, which is in line with the results of immune-detection of CMV 1a and 2a proteins in CMV-infected protoplasts. Thus, we have made the necessary modifications to the viral replication compartment, in which the relative concentrations of CMV 1a and 2a proteins are adjusted (please see Fig 10 in the revised version). We hope that this revised figure better reflects the actual replication compartment of CMV.

B. In early work in the late 70’s in the lab of J.M. Kaper, it was shown that when a satRNA was replicated in tobacco, the satRNA accumulated to such a high level (as did its dsRNA) that the level of helper virus was greatly suppressed. This did not happen in squash, where there was little suppression of helper virus with moderate levels of satRNA accumulation and no high levels of satellite dsRNA. Thus, the host can influence the replication levels as much as anything else! In fact, those workers often found satRNAs in field isolates, but only after passaging them in tobacco! Thus, very low levels of satellite can be amplified under specific circumstances.

C. Also pertinent to the work here is a study from the Bruening lab, in which they found that trace amounts of TRSV-satRNA could be stimulated to replicate in the presence of non-accumulating satRNA mutants. [Virology (1995) 209: 470-479.]

D. SatRNAs also can be amplified by CMV and persist in a fungus and in an oomycete! [Eur. J. Plant Pathol. 2019, 153: 1001-1017.] This indicates that the host requirements for basic replication are minimal and likely ubiquitous.

E. It may be that the LS-CMV replicase is not as efficient as the Fny-CMV replicase. Alternatively, it may be that the LS-CMV 1a and 2a proteins are not as good at suppressing hosts defenses against the accumulation of the virus as are the Fny-CMV 1a and 2a proteins. In the former case, this then comes down to an issue of Km of the replicase for interaction with the satRNA. However, the agroinfiltrated satRNAs are not present at 0.1 fg / microgram of (virion) RNA, as were the endogenous TRSV-satRNA. In addition, while the mixed strain replicases might be less efficient than the parental virus replicases, since the 1a and 2a proteins from different strains are not adapted to each other, this is actually not the case here. Rather, both the 1a and the 2a of Fny-CMV contribute separately to the efficiency of RNA3 replication and of satRNA replication, with the LS-CMV 1a and 2a together showing the least efficiency (or most turnover).
In any event, the authors should consider these points to decide what the most likely scenario is.

**RE:** Integrated responses to these points B-E. Thanks so much for providing these important points for us to consider the possible scenario. We have considered these point carefully, especially the work on TRSV-satRNA, and the importance of hosts on satRNA replication efficiency. Thus, we discussed the similar scenario on TRSV-satRNA reported by the Bruening lab, and the importance of hosts in amplifying viral RNAs in the Discussion section as follows: “A similar scenario was reported previously about the tobacco ringspot virus-associated endogenous satRNA, which is latently presence, but can be stimulated by two non-accumulating satRNA mutants [72]. It is still unknown how the non-accumulating satRNA mutants function in this event. Our data clearly demonstrates that CMV RNAs rely on their recruitment and replication for the stimulated replication of satRNAs (Figs 5-6 & 8-9). In addition, plant hosts play a vital role as well in the replication levels of satRNAs as demonstrated by the early work showing that CMV satRNAs accumulate at a limited level in squash, but reach to a high level in tobacco [73]. Moreover, they found the existence of satRNAs in major proportion of field CMV isolates only after passaging them in tobacco. Thus, very low levels of CMV satRNAs can be amplified under specific circumstances. Combined with our finding, CMV RNAs would work together with host factors to determine the replication level of satRNAs in host plants.” (lines 584-596).

Yes, we agree with the reviewer that the defect of the replicase of LS-CMV could be due to its relatively weak activity in amplifying viral RNAs and satRNAs. We think the possibility of the difference in suppressing host defense because the RNA silencing suppressor activity was included in all the trans-replication assays conducted in this work as we mentioned in the manuscript as follows: The difference in producing RNA3 and RNA4 between both viral replicases would be due to the distinct replication efficiency, because the RNA silencing suppressor p19 was included to suppress antiviral RNA silencing in this assay (lines 289-292). Meanwhile, we discussed the possible reasons in the Discussion section as follows: “Interestingly, we found that the replicase of LS-CMV is defective at replicating satRNAs when viral RNAs are absent. Our data hints two causes for the defect. One is the weak replication activity, as evidenced by the results showing that the replicase of LS-CMV is not proficient as the replicase of Fny-CMV in amplifying RNA3s of both CMV strains (Figs 1&7). The second is the poor capability of recruiting the (+) strand of satRNAs (Figs 8). These causes are consistent with our finding that both the 1a and 2a of Fny-CMV contribute separately to the efficiency of RNA3 replication, and of satRNA replication (Fig 7), because CMV 1a and 2a are involved in RNA recruitment and RNA replication, respectively.” (lines 616-624).

**Reviewer #2:** The ms focuses on the role of the helper virus in the replication of satellite RNA using two strains of cucumber mosaic virus (fny and LS) that show different ability to support replication of its associated satellite Sat-1 RNA as model system. The authors have established a nice in trans replication assays to dissect the different components of the help virus that is needed for sat-1 replication. Using this system, they initially established that the fny1 replication proteins and not those of LS can support sat-1 replication and that the addition of the viral RNAs is sufficient to stimulate sat-1 replication. The topic holds significant interest as it advances our understanding on RNA sequences and protein dependence between the satellite RNA and its helper virus. The ms is well written, the experiments are nicely laid out. However, some additional controls are needed to provide support of their conclusions.

**RE:** Thank the reviewer’s positive feedback, and we appreciate very much for your time. We have added all controls you requested, and incorporated the results in the revised version.

Reviewer #3: The primary objective of this study is to explore the non-templated functions of genomic RNA derived from the Cucumber mosaic virus (CMV) in the amplification of satellite RNA (sat-RNA). To achieve this objective, the researchers utilized two distinct CMV strains, CMV-Fny and CMV-LS, belonging to subgroup I and II, respectively. Additionally, the study assessed the replication efficiency of various sat-RNAs in the presence of CMV strains Fny and LS, as well as mutant sequences. The study's findings led to the conclusion that non-templated functions of CMV RNAs play a crucial role in sat-RNA replication. Unfortunately, as elaborated below, this reviewer disagrees with the conclusions, as the authors have omitted critical controls and key experiments necessary to substantiate their claims.

**RE:** Thank you for your time, and raising constructive comments. We have tried our best to include the controls as you requested. We hope you will be satisfied with the revised version.

**Part II – Major Issues: Key Experiments Required for Acceptance**
Please use this section to detail the key new experiments or modifications of existing experiments that should be absolutely required to validate study conclusions.

Generally, there should be no more than 3 such required experiments or major modifications for a "Major Revision" recommendation. If more than 3 experiments are necessary to validate the study conclusions, then you are encouraged to recommend "Reject".

Reviewer #1: N/A

Reviewer #2: Main comments:
The authors revealed that the non-coding version of the LS genomic RNAs are sufficient to support sat-1 replication in their trans- replication assay. However, they missed to establish sequence specificity. Could an unrelated non-coding viral sequence or non-coding Fny viral RNAs work as well in complementation with the LS replication proteins? How to explain the inability of ncL3 to support D4 replication?

**RE:** Thanks for raising the point about the sequence specificity. We have added the work on the non-coding RNAs of Fny-CMV and another cucumovirus tomato aspermy virus in Fig 2D & E. The results show that all of these non-coding RNAs stimulate the replication of sat-T1, demonstrating that the stimulation of sat-T1 replication by viral RNAs is not strictly dependent on viral RNA sequences. By the way, we removed the data (Fig 2D in the original version) about the work on the coding RNAs of Fny-CMV, because it does not provide any useful information any more. Indeed, there is an obvious variation in stimulating replication of various satRNAs by ncL3, some satRNAs accumulate at a relative low level, which could be invisible. But in the new data repeated with additional controls (Fig 3A), the positive-sense and minus-sense RNAs of sat-D4 and other satRNAs were detected.


The authors concluded that the LS genomic RNAs must be a replicative RNA to support sat-1 replication. However, their approaches are not “direct” and the provided data are not strong enough to fully rule out that a non-replicative RNA could still support sat-1 replication. In fact, the deletion or mutation of the TSL, 5’UTR or the box-B could result in RNA instability more than a direct interference with the RNA replication. The authors should find other ways to rule out that a non-replicative RNA is indeed non-functional for sat-1 replication: how about adding just “TLS” in trans? could the TLS alone be sufficient to support sat-1 replication? How about using sgRNA 4 as viral RNA (its non-coding version) since sgRNA 4 is non-replicative and it shares the same 3’ UTR as the gRNAs? Another option is to repeat the assay in an in-vitro replication system (as referred by the authors in their introduction). In such system, the TLS mutant and deletion mutant RNAs etc could be stable enough to measure sat-1 replication in a short time frame.

**RE:** Thanks for the comment. We have added the work on the effect of the TLS, RNA4 and its noncoding RNA on the replication of sat-T1 in the presence of the replicase of LS-CMV. As shown in Fig 4E, all of these RNAs are dysfunctional in stimulating sat-T1 replication. The results have been described in the revised manuscript as follows: “When the TLS was provided *in trans* in the replication assay, sat-T1 replication was not stimulated (Fig 4E). Moreover, when the TLS was provided in RNA4 (L4) or its non-coding RNA (ncL4) of LS-CMV, both viral RNAs did not work either in stimulating sat-T1 replication, as did the TLS itself (Fig 4E). These results clearly demonstrate that the TLS is essential, but not sufficient for viral RNAs to stimulate sat-T1 replication by LS replicase.” (line 397-342).

In Fig 7, the authors concluded that Ls1 is partially responsible for the defect of LS replication protein in supporting sat-1 replication. However could it be that LS1a did not support sat-1 replication because it is not compatible to form a complex with F2a? The authors should complement their experiment by looking at the ability of Ls1a+F2a (and F1a+Ls2a) to support sat-1 replication in the presence of the viral RNAs.

**RE:** Yes, we have done the experiment as requested. The data has been shown in Fig 7C. The results are described as follows in the manuscript: “When L3 was co-expressed with sat-T1, the heterologous combination F1a + L2a was closely efficient as F1a + F2a in the amplification of the (+) strands of sat-T1, albeit of much less efficiency to build up the (-) strands. Compared with L1a + L2a, the heterologous replicase L1a + F2a was even less efficient in amplifying (+)-stranded sat-T1, while exhibited similar ability to synthesize (-) strands (Fig 7C). These results indicate both heterologous replicases (F1a + L2a, L1a + F2a) were adaptable in replicating viral RNAs, and satRNAs when viral RNAs are present, in spite of the varied efficiencies.”(line 484-491).

Line 430-460: the authors should include some binding assays or co-localization assay to support their conclusion of inability of the LS replicate to recruit satRNAs.

**RE:** Yes, we developed a bacteriophage MS2 CP-RNA binding-based methodology, which showed the colocalization of Fny 1a, but not LS 1a, with sat-T1 in nuclei, as shown in Fig. 9. These results support our inclusion of inability of the LS replicase to recruit satRNAs. The results are described in lines 529-552.

Reviewer #3: Before embarking on the planned experiments aimed at addressing the research questions, the authors should lay the groundwork by examining the accumulation levels of sat-RNA in the presence and absence of each HV strain. Additionally, it is imperative to consistently evaluate the levels of both plus and minus strands of sat-RNA accumulation in every experiment.

**RE:** Thanks for the comment. We have added the experiments on the replication of sat-T1 in the presence and absence of each CMV strain in Fig 1A. Both CMV strains show nearly equal ability in boosting sat-T1, while sat-T1 is undetectable in the absence of each CMV strain, even overexposed. And, minus strands of sat-RNA accumulation were added into every experiments, except for Figs 8 & 9. In most cases, minus-strands of satRNAs were detected when positive-strands are present in the same samples. Interestingly, we found that the replicase of Fny-CMV, but not the replicase of LS-CMV produce minus-stranded dimers of all satRNA strains tested in the trans-replication assays (Figs. 1C & D, Fig 3). We discussed its potential in the replication of satRNAs in Discussion section (line 636-652).

#1. Four decades ago, it was established that in the absence of its helper virus (HV), satellite RNAs (sat-RNAs) can endure for up to 25 days, as documented in Virology (94: 243-253) and Virology (86: 562-566). The molecular basis underlying the HV-independent long-term survival of sat-RNA was attributed to its ability to localize within the nucleus and exploit host enzyme activities, akin to viroids and Hepatitis delta virus (J Virology 86: 4823-32). In light of these findings, it is crucial for any study involving sat-RNA replication to incorporate a control group that solely inoculates sat-RNA and assesses the accumulation of both plus and minus strands. Notably, this vital control is conspicuously absent in all experiments conducted in this study.

**RE:** Thanks for this comment. I agree it was our ignorance. Hope our responses to this comment are reasonably acceptable. I will be totally open to any more comments on this issue. Thanks! We have carefully considered the scenario of satellite RNA (satRNA) long-term survival independent of the virus. Therefore, we included the control expressing satRNA alone in Figures 1A, 1C, and Figure 3. Unfortunately, satellite RNAs in this control is undetectable, even though overexposed, indicating that the accumulation of satellite RNAs solely in the plant is below the detection sensitivity of our detection method. This is consistent with the results we reported earlier (Nucleic Acids Res. 2019; 47(15):8255-71). Similarly, even in the presence of the LS replication proteins, satRNAs could not be detected either. By comparing our detection method with the one reported successful in detecting helper-virus-independent satRNAs, we identified some important differential parameters, including probe labeling methods (digoxin vs isotope P32), the amount of total RNA loaded (2 micrograms vs 20 micrograms), and the plants used (*N. benthamiana* vs *N. clevelandii*). These differences provide a reasonable explanation for the discrepancy in the detection of satRNAs in the absence of CMV. In other words, in our experiments, all satellite RNAs detected, including their positive and negative strands, are believed to be the products resulting from effective replication by the virus replicase. **We have explained this situation in the discussion section.** Despite the limitations in the detection sensitivity of our method, we believe it does not affect the accuracy of our experimental results and the reliability of our conclusions.

Additionally, concerning the experimental control, as mentioned earlier, we have incorporated the control with solely expressing satRNA in some experiments. However, in other experiments, we did not include this control. Our reasoning is as follows: in these other experiments, our starting point is based on the defect of the LS replication proteins in replicating satRNA. Therefore, this treatment (L1a+L2a+satRNA) logically serves as the experimental control. Even if the control solely expressing satRNA was included in these experiments, it would not impact the results. In addition, in Fig 1E, we did not include this control because the results involve the accumulation of viral and satellite RNAs in upper systemic leaves, thus, it is not necessary to consider Agrobacterium-mediated satellite RNA transcription.

#2. The data displayed in Figure 7A should be integrated into Figure 1 to assess the relative replication competence of RNA3 in the presence of heterologous RNAs 1 and 2, specifically, F1a+F2a+L3 and L1a+L2a+F3. Additionally, the authors should provide an explanation for why L3 exhibits more efficient replication with F1a+F2a compared to its homologous counterpart, L1a+L2a. Compare RNA3 and 4 levels Fig. 1A and 7A.

**RE:** Yes, we added the work on the replication of F3 and L3 assisted by F1a+F2a or L1a+L2a in Fig 2B. It clearly shows that F1a+F2a exhibits equally efficient ability to replicate F3 and L3, which is much higher than L1a+L2a. This suggests that F1a+F2a possesses stronger replication ability no matter in the presence of the homologous or heterologous RNA3. In another words, the replicases of Fny-CMV and LS-CMV are totally compatible with the heterologous RNA3. This has been explained in the revised manuscript as follows: “The difference in producing RNA3 and RNA4 between both viral replicases would be due to the distinct replication efficiency, because the RNA silencing suppressor p19 was included to suppress antiviral RNA silencing in this assay.” (line 289-292).

#3. The data presented in Figure 2 (B, D) unmistakably demonstrates that the presence of CP (comparing L3 in Figure 2B to lane ncl3 in Figure 2D) plays a substantial role in boosting sat-RNA replication. In positive-strand RNA viruses, CP has been well-documented as a regulator of strand asymmetry (as discussed in Ann Rev Phytopath 43:39-62 and Phytopathology 110: 228-236). Consequently, to verify this possibility, it is imperative to conduct an experiment that involves co-expressing CP with the inoculum as depicted in Figure 2D, lane ncL3.

**RE:** Thanks. We have added this work as requested, the data is shown in S1 Fig. Indeed, providing CP *in trans* increases the accumulation of ncL3, but also sat-T1, as described in the revised manuscript “By comparing the efficiencies of L3 and ncL3 in stimulating sat-T1 replication (Figs 2B & C), it seems that L3 was more efficient than ncL3. In some (+)-strand RNA viruses, CP has been well-documented as a regulator of strand asymmetry [60, 61]. Indeed, when compared with co-expression of green fluorescence protein (GFP), co-expression of LS-encoded CP protein with ncL3 increased the accumulation levels of ncL3 and both polarities of sat-T1 by 23%-67%, when combined with the LS replicase, but the asymmetry of sat-T1 replication was not changed much (S1 Fig).” (line 332-339)

#4. The data presented in Figure 3B appears to be puzzling. It's unclear why all lanes (excluding the vector lane) that do not contain satRNA are showing the presence of satRNA.

**RE:** We are sorry for the misleading. (-) denotes the absence of ncF3, rather than the absence of satRNA. In the revised version, we have changed the style to show the figure, which would be clear to follow up.

#5. In this study, the authors employed DIG-labeled DNA probes. While DIG-labeled probes offer safety and convenience, they may not be suitable for longer exposures of the blot. It is advisable to opt for radio-labeled RNA probes when extended exposures are necessary to detect poorly accumulated satRNAs and require high-stringency post-hybridization washing.

**RE:** Thanks for the advice. At moment, Dig-label is the best choice for us, I think, because the isotope ^32^P is not available in the mainland, China, and any order from abroad is strictly managed and controlled. So, we are so sorry not to take the advice.

• Other major issues:

1. Throughout the manuscript, the authors consistently use the term "infection" (e.g., in lane 264, 267). It's important to note that replication and infection represent two distinct phases in the life cycle of an RNA virus. In contrast to replication, infection is a multifaceted process encompassing replication, movement, and symptom development. In this study, the authors exclusively assessed replication and did not examine the broader concept of infection. It is essential to rectify this terminology throughout the manuscript to accurately reflect the scope of the research.

**RE:** Thanks for clarifying the terms. We have checked this throughout the manuscript and made corrections.

2. The accumulation levels of satRNA-T1 in the presence of F1a+F2a varied significantly between experiments. Compare Fig. 1C3B. Why?

**RE:** This is caused by the different exposure time. In Fig 3B, we did not expose the membrane to X-ray films too long, because too long time would lead to the presence of some over-exposed bands in the presence of ncF3. Short time exposure would give us better idea about the stimulation of satRNA replication by the presence of ncF3.

3. Unless the outcomes of the experiments recommended above are taken into consideration, this reviewer contends that the data presented in Figures 4-8 lack relevance.

**RE:** We hope the outcomes of these experiments we added would be taken into consideration.

4. This reviewer encountered difficulty in comprehending the model depicted in Figure 9. For instance, positive-strand RNA viruses, such as CMV, typically replicate within vesicles or spherules induced by the replicase protein of the helper virus (HV) (as discussed in Virology 383: 248-260). In the top panel of Figure 9, it remains unclear how VRC (Virus Replication Complex) formation can occur in the absence of HV RNAs. This aspect requires clarification.

**RE:** We are sorry for not making the model clear. In fact, recently, Wang and colleagues (J Integr Plant Biol. 2021;63(2):353-64) reported CMV replicases themselves can establish viral replication compartment-like spherules in plant cells, which is independent of viral RNAs. So, we have added this information in the Discussion (line 605-607) and in the legend of Fig 10 showing the working model.

5. Discussion: This section needs to be rewritten. Many of the conclusions drawn in this study rely on the results of experiments conducted in the absence of viral RNAs. However, as previously mentioned, the authors failed to evaluate the accumulation of both plus- and minus-strands of satRNA in the absence of the HV strain. Without this essential information, it becomes challenging to critically assess the non-templated functions of HV RNAs.

**RE:** We have rewritten the Discussion section, and we hope this one is more informative and relevant.

**Part III – Minor Issues: Editorial and Data Presentation Modifications**
Please use this section for editorial suggestions as well as relatively minor modifications of existing data that would enhance clarity.

Reviewer #1: Other specific issues:
1. ln 88-98. Delete “on the other hand”, since it is the same in this respect to RNA2, both of which have two ORFs.

RE: Done

2. ln 175 and 197. Italicize “Agrobacterium”.

RE: Done, also corrected the same issue somewhere else.
3. ln 195. Change agrobacterium to Agrobacterium.

RE: Done, also corrected the same issue somewhere else.
4. ln 312. Change “PITs” to “PISs”?

RE: It is not present in the manuscript any more.
5. ln 321. Change S2D to S2C.

RE: The S2 Fig has been removed.
6. ln 375. Change Fig 6A to Fig 5A.

RE: Done
7. ln 381. Change Fig 6B to Fig 5B.

RE: Done
8. ln 383. Change Fig 6C to Fig 5C.

RE: Done
9. ln 419. 42 vs. 13 is more than 3-fold and not just 2-fold.

RE: Thanks, corrected.
10. ln 426-428. The conclusion is accurate, but the heading of this section (ln 409-410) is not, as is also the case for the statement on the next page (ln 432-433)!

RE: Thanks, both the heading and the statement are corrected accordingly.
11. ln 454, change from 58% to 68%, as given in Fig 8C, lower blot.

RE: Thanks, corrected.
12. ln 485-488. There may be many more places in the RNAs of the helper virus that satRNAs can interact with than just the two the authors tested. [See Nucleic Acids Res. (1986) 14: [3229-3239](tel:3229-3239), and Physiol. Mol. Plant Pathol. (1993) 42: 195-204.] The latter paper also showed that interactions with LS-CMV (then known as L2-CMV) and another subgroup II strain, WL-CMV, by several satRNAs, were less efficient at interacting than for other CMV strains.

RE: Thanks for providing the extra information. As requested by another reviewer, it is suggested to remove this section about the potential interaction between viral RNA3 and satellite RNA. We agree with the reviewer’s comment because it is still not clear the importance of the trans base-paring between RNA3 and satellite RNA. So, we have removed this section in this revised version.

13. In the M & M section, it states that the (+)and (-) T-sat RNA insertions in the Box-B modified Fny-CMV RNA 3 (mF3) were done downstream of the CP, as depicted in Fig. 8, and this was verified in the Results section (ln 444). However, on ln 525-526, it is written ambiguously, and it can be read that the T1-satRNA sequence was inserted INTO the modified Box-B sequence, rather than downstream at the beginning of the 3’ UTR. This should be corrected. It would have been interesting to see if the Box-B motif could have been replaced directly with the satRNA!

RE: Thank you for pointing out this issue. The words on ln 525-526 have been removed in this revised version, since we rewritten the Discussion section as required by one of reviewers. But, we checked somewhere else, to avoid the same issue in this manuscript. Yes, initially, we tried to replace the Box-B sequence with sat-T1 in F3 and L3, but the replacement made both R3 recombinants lethal when combined with the replicase of LS-CMV. We have included this information in S4 Fig in the revised version with the words “Initially, we found that substitution of the Box-B sequence in either F3 or L3 with (+)-strand of sat-T1 was detrimental to the variants of both RNA3s when combined with the replicase of LS-CMV (S4 Fig).”(line 508-510).

Reviewer #2: Minor comments:
Fig 2. How to explain the low level of sat-1 replication in the 2b-lacking viral RNAs?
RE: the least capability of L2Δ2b to stimulate sat-T1 replication could be due to over-production of the 2a protein translated from the replicable RNA2 mutant, which might disrupt the balance between the 1a and 2a proteins during replication. The words have been added in the revised version on line 329-332.

Line 298-322: this section can be deleted since it is not even clear why would one assume the importance of in trans base pairing.

RE: Thanks for the suggestion, and we agree with you that it is still not clear about the importance of in trans base paring between viral and satellite RNAs. We have removed this section, the S2 Fig and related information in the Discussion section from the revised version.

Line 334: it should neither MP or CP “coding RNA sequences”

RE: Yes, we have corrected it to “the coding sequences of MP and CP are not essential for…”

Reviewer #3: 1. L27 and several other places: “recruit” to where. Clarify
2. L35: “subviruses”. Sat-RNA are not viruses. They are rereferred to as “subviral pathogens” not “subviruses”. Correct

RE: Done
3. L59: Pivotal questions: Like what, give examples.

RE: Yes, it is done as showing “Despite substantial achievements have been made already [2-4, 8-14], some pivotal questions surrounding satRNAs remain unanswered, such as biologically-supported RNA structure and its interplay with viral proteins” on line 59-61.

4. L104-106” “..that viral RNAs compete…..decreased replication of levels of satRNAs. This assertion appears to be inaccurate. The available evidence suggests that sat-RNAs, which are typically less than 300 nucleotides in length, effectively hijack the viral replicase and competitively outperform longer viral RNAs. Consequently, sat-RNAs tend to accumulate at levels several times higher than those of the helper virus (HV) RNAs.

RE: Thanks for the discussion. This sentence is just the objective statement of the result that Wu and colleagues obtained in vitro (Res Virol. 1995;146(1):61-7.). I agree with you that satRNAs undoubtedly are successful in competing for viral replicase with viral RNAs. So, following the sentence, we added the words “In fact, CMV satRNAs usurp viral replicases effectively for their replication, leading to a high accumulation level. This hints that satRNAs possesses potential mechanisms to prompt their replication.” (line 108-110). Hope this change makes it acceptable.

5. L106-107: “Thus it is widely accepted that helper virus RNAs and satRNAs mutually impede each other’s replication”. To substantiate this conclusion, the authors should consider providing a reference from the scientific literature that supports this widely accepted concept in virology.

RE: Combine with the comment 4 above, we think it is more acceptable to rephrase this sentence to be “In fact, CMV satRNAs usurp viral replicases effectively for their replication, leading to a high accumulation level. This hints that satRNAs possesses potential mechanisms to prompt their replication.” (line 108-110)” . Meanwhile, we removed the similar statement from the Abstract.

6. L113-116: Either CMV RNAs….CMV strain. Does not make any sense. Please reword for clarity.

RE: L113, “Either CMV RNAs or their mutants” has been reworded to “viral genomic RNAs”. L116, “either CMV strain” has been reworded to “Fny-CMV or LS-CMV”.

7. L271: “that that”. Edit

RE: Done
